# Long-Range Self-Hybridized Exciton-Polaritons in Two-Dimensional Ruddlesden–Popper Perovskites

**DOI:** 10.1021/acsphotonics.4c00824

**Published:** 2024-07-31

**Authors:** Maximilian Black, Mehdi Asadi, Parsa Darman, Sezer Seçkin, Finja Schillmöller, Tobias A. F. König, Sara Darbari, Nahid Talebi

**Affiliations:** † Institute of Experimental and Applied Physics, Kiel University, Kiel 24098, Germany; ‡ Nano-Sensors and Detectors Lab., Faculty of Electrical and Computer Engineering, Tarbiat Modares University, Tehran 1411713116, Iran; § Leibniz-Institut für Polymerforschung Dresden e.V., Dresden 01069, Germany; ∥ Center for Advancing Electronics Dresden (cfaed), Technische Universität Dresden, Dresden 01062, Germany; ⊥ Faculty of Chemistry and Food Chemistry, Technische Universität Dresden, Dresden 01069, Germany; # Kiel Nano, Surface, and Interface Science KiNSIS, Kiel University, Kiel 24118, Germany

**Keywords:** exciton-polariton, 2D Ruddlesden−Popper perovskite, self-hybridized, strong coupling

## Abstract

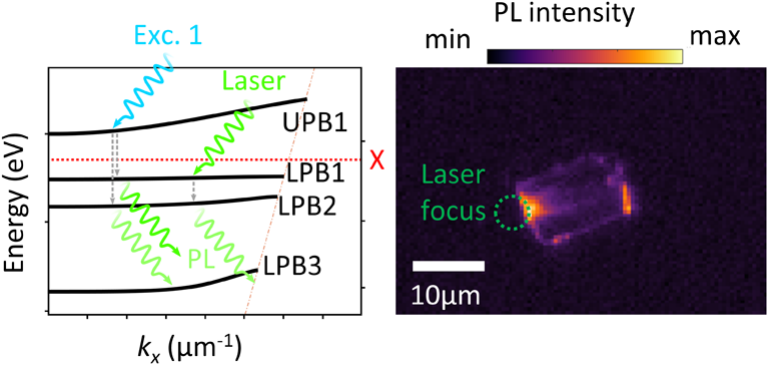

Lead halide perovskites have emerged as platforms for exciton-polaritonic studies at room temperature, thanks to their excellent photoluminescence efficiency and synthetic versatility. In this work, we find proof of strong exciton–photon coupling in cavities formed by the layered crystals themselves, a phenomenon known as the self-hybridization effect. We use multilayers of high-quality Ruddlesden–Popper perovskites in their 2D crystalline form, benefiting from their quantum-well excitonic resonances and the strong Fabry–Pérot cavity modes resulting from the total internal reflection at their smooth surfaces. Optical spectroscopy reveals bending of the cavity modes typical for exciton-polariton formation, and absorption and photoluminescence spectroscopy shows splitting of the excitonic resonance and thickness-dependent peak positions. Strikingly, local optical excitation with energy below the excitonic resonance of the flakes in photoluminescence measurements unveils the coupling of light to in-plane polaritonic modes with directed propagation. These exciton-polaritons exhibit high coupling efficiencies and extremely low loss propagation mechanisms, which are confirmed by finite difference time domain simulations. Thus, we prove that mesoscopic 2D Ruddlesden–Popper perovskite flakes represent an effective but simple system to study the rich physics of exciton-polaritons at room temperature.

## Introduction

Strong light–matter interactions are central to many physical and chemical phenomena, ranging from the coherent control of chemical reactions to the formation of new states of matter. Being first described as superpositions of light and matter excitations,^1,2^ polaritons form when an elementary material excitation interacts strongly with an electromagnetic radiation field. Most notably, excitons in a semiconductor can strongly interact with confined light, such as the resonant modes of a cavity, and the resulting quasi-particle is hence called exciton-polariton (E-P). E-Ps exhibit exceptional properties such as low effective mass, long coherence lengths, and enhanced nonlinearities,^3^ opening up possibilities to study effective photon–photon interactions leading to Bose–Einstein condensation^4,5^ (BEC) and many-body states in the strong-coupling regime.^6,7^ However, it is challenging to transfer the strong nonlinearities and subsequent polariton condensation to room temperature. Therefore, it is necessary to use the control parameter of E-P density as a substitute for the temperature, expressing the need for high exciton yield semiconductors with sufficiently strong exciton binding energies. To realize E-Ps, a variety of semiconductors have been combined with different ways to confine light and to achieve overlaps with excitonic wave functions. Prominently, transition-metal dichalcogenides (TMDCs) have been used because of their exceptionally high exciton binding energies of a few hundred electronvolts and high oscillator strengths for effective light–matter coupling, particularly in the case of monolayers.^8,9^ In practice, TMDC monolayers have been merged with high-quality microcavities,^10^ photonic and plasmonic crystals,^11^ plasmonic nanoantennas,^12^ and plasmon polaritons.^13^ Recently, layered structures consisting of alternating TMDC monolayers and dielectric insulators,^14^ as well as chemical vapor deposition grown TMDC monolayers on truncated all-dielectric photonic structures yielding Bloch surface waves,^15^ have demonstrated strong coupling and long polariton propagation. Yet, the handling of TMDC monolayers proves to be quite challenging, and they barely reach lateral dimensions exceeding several tens of micrometers when obtained by mechanical exfoliation. Additionally, deposition methods for a large-area monolayer typically exhibit grain boundaries and nonuniform crystallinity.

Lead-halide perovskites have proven to be a suitable alternative due to their remarkably easy synthesis, direct band gaps, high optical gain, and high oscillator strength.^16,17^ In particular, of special interest are quasi-two-dimensional Ruddlesden–Popper organo-metal halide perovskite (RPP) layers that have been applied in solar cells in 2015^18^ and have been used for realizations of photodetectors, LEDs, and lasers.^19−21^ These layers are quantum-well-structured materials of a certain number of perovskite layers (well), which are separated by spacer molecules (barrier) along the *z* axis.^22−24^ For these quasi-two-dimensional materials, exceptionally high exciton binding energies of several hundred millielectronvolts have been reported in bulk crystals^25−28^ and connected to quantum confinement, decreasing the likelihood of electron–hole pairs dissociating at charge separation interfaces, which additionally results in a high emission quantum yield. For perovskites, polariton formation has been realized with a variety of photonic structures,^29^ such as plasmonic lattices^30^ and microcavities,^31−33^ with superfluidity claimed to be reached in the latter systems.^34^ Perovskite nanoplatelets in photonic cavities have been reported to exhibit polaritonic lasing,^35^ while RPP platelets in a similar cavity have been documented to show two power thresholds.^36^ Furthermore, polariton condensation has been announced in perovskites synthesized within high-quality microcavities followed by the formation of XY Hamiltonian lattices facilitated by reflective spatial light modulators.^37,38^ Additionally, in a similar system incorporating perovskites in a Hall lattice design, a propagating topological edge state with polariton condensation has been demonstrated.^39^ Although high-quality optical cavities incorporating distributed Bragg reflectors provide high tunability of the cavity resonances to the excitonic energies, their complex fabrication schemes hinder their implementation into versatile optoelectronic devices. In addition, plasmons provide an evanescent electromagnetic field close to metal-dielectric interfaces that can strongly interact with excitons especially when localized plasmons or plasmonic lattices with tunable collective lattice resonances are involved.^40^ Nevertheless, plasmons sustain significant dissipative losses, which necessitate precise nanofabrication processes. Therefore, there is a fundamental need for simple and efficient platforms that enable robust exciton–photon coupling.

Recently, waveguide modes of thin semiconductor crystals strongly interacting with excitons have gained interest in a process called self-hybridization. TMDC layers of tens of nanometers in thickness have been studied by optical microspectroscopy^41,42^ and scanning near-field optical microscopy,^43^ where both exciton–photon anticrossing behavior and E-P propagation have been detected.^44^ Additionally, using cathodoluminescence spectroscopy, where the confined Cherenkov radiation strongly couples to the excitons,^45^ propagation mechanisms of E-Ps with high spatial resolution, edge E-Ps, as well as polariton–polariton interactions were explored.^46^ Furthermore, it has been shown that the energy band gap of plasmonic crystals can be tuned by combining the thin TMDC layers with plasmonic crystals due to the strong coupling of exciton-polaritons with the plasmonic Bloch modes.^47,48^

Whereas strong luminescence from excitons has been observed only for mono- and bilayer TMDCs, excitons in bulk RPP crystals retain their 2D properties of high binding energies and prominent luminescence peaks. Large single-crystal flakes of quasi-two-dimensional perovskites have been shown to feature polariton-related bending of optical cavity modes in their reflection spectra^49^ and spin-dependent exciton–exciton interactions.^50^ Furthermore, energy transfer from higher- to lower-energy E-Ps has been shown in RPPs on gold substrates by direct laser excitation of lower polariton branches and ultrafast spectroscopy.^51,52^ However, direct proof of propagating exciton polaritons in freestanding thin RPP crystals and an extensive investigation of their propagation properties and length have yet to be reported.

In this work, we investigate the optical properties of thin exfoliated RPP flakes by means of photoluminescence (PL), reflection, and absorption spectroscopy combined with analytical and finite element calculations of the layered system. We find a clear anticrossing of cavity modes in the vicinity of the exciton energy in reflection and absorption spectra in excellent agreement with our theoretical results. By applying site-selective PL excitation of lower polariton branches and coupling out of E-Ps at the edges of our flakes, we confirm the long-range propagation of polaritons across the waveguide. These findings are confirmed with finite-difference time-domain simulations predicting extremely low loss propagating waves at the measured energies. Our results prove that even simple quasi-confined modes of a two-dimensional RPP waveguide coupled to excitons yield high-quality E-Ps at room temperature. This will spark further investigations utilizing the intriguing fabrication simplicity toward BEC in these straightforward systems.

## Results

**Figure 1:**
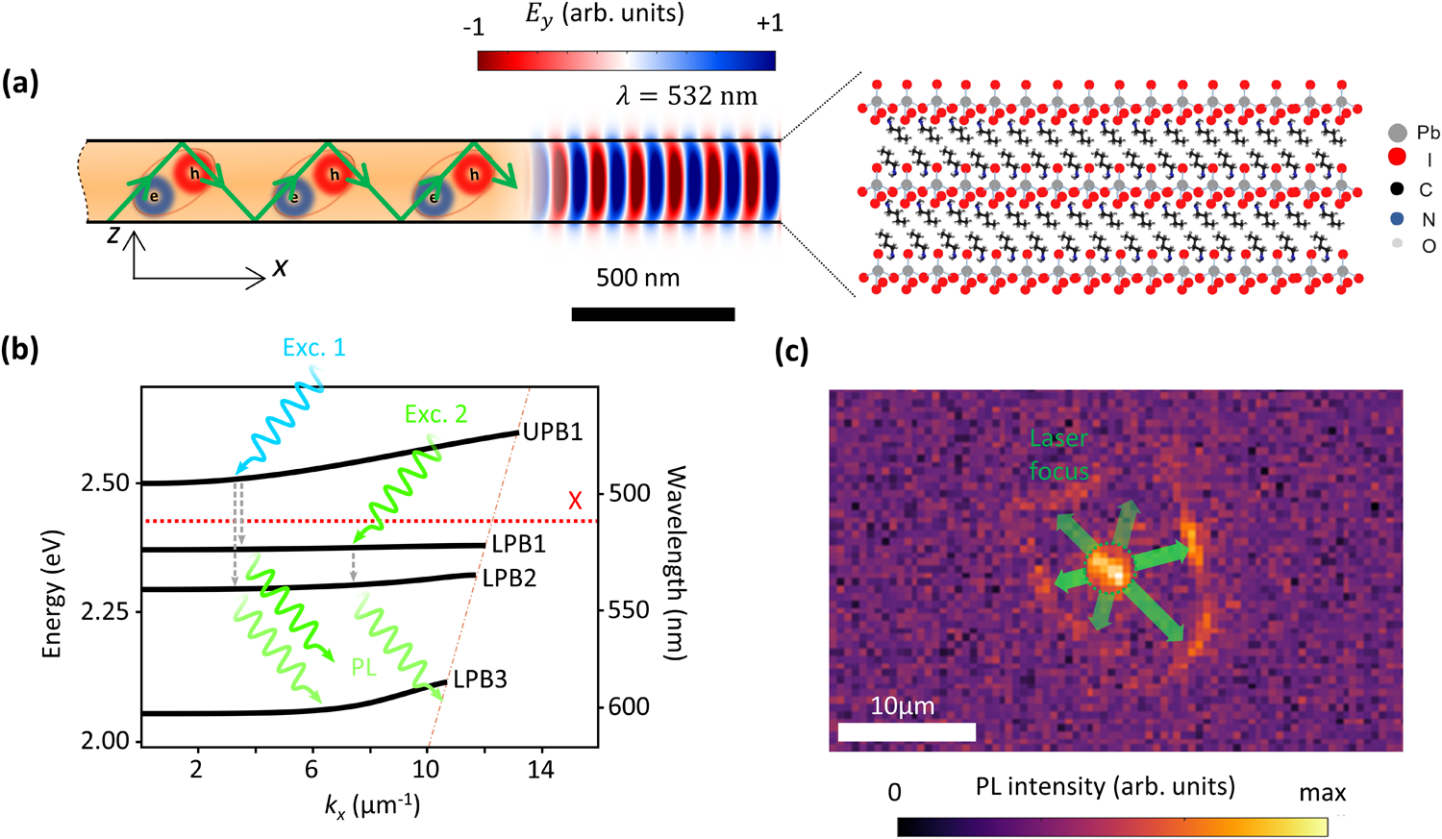
(a) Self-hybridized exciton-polaritons in thin RPP crystals. Excitons supported by the stacked quantum-well structure of the RPP depicted on the right side interact with the waveguide modes that stem from reflection at the boundaries of the thin film, leading to polariton formation. As an example, the *y-*component of the electric field of the resulting first-order even mode is shown. (b) Excitation and radiation schemes of the measured photoluminescence (PL) signal. Light with an energy lower or higher than the exciton resonance couples to lower or upper polariton branches (LPB and UPB), respectively. The excited states undergo relaxation processes to the LPBs situated below via either nonradiative or radiative paths, where the emission within the infrared to the visible range is detected (green wiggly arrows pointing to the bottom-right). The LPB coupling to light causes a detectable PL signal. (c) PL intensity map of an RPP flake excited by a 532 nm CW laser focused at its center. Excited lower polariton modes propagate to the edges of the flake, where they couple to light.

We mechanically exfoliate submicrometer thin flakes of two-dimensional Ruddlesden–Popper perovskites (2D RPP) from macroscopic sheets of bulk crystals onto a glass substrate. The bulk RPP (BA)_2_PbI_4_ is grown in a solution, and its chemical structure is schematically depicted in Figure 1a, showing its layered configuration of monolayers of the metal-halide PbI_4_^2–^ which are separated by butylammonium cations (BA^+^). The monolayers themselves consist of octahedra formed by lead(II) cations surrounded by six iodide anions. We note that we use single atomic layers (*n* = 1) of these octahedra, which implies the absence of anions that stabilize the octahedra found in the crystal structure of multilayer (*n* > 1) 2D RPPs or 3D perovskites.^29^ The monolayers host excitonic excitations, which exhibit quantum confinement as a result of being sandwiched by organic cations, consequently resulting in high binding energies of the excitons. Additionally, the structure of such stacked quantum wells enables the mechanical exfoliation of the bulk crystal to submicrometer thin flakes. Moreover, the dielectric constant of the barrier (BA^+^) is less than that of the inorganic well (PbI_4_^2–^), which contributes to high exciton binding energies according to dielectric confinement phenomena. Aliphatic molecules such as BA have been shown to have greater exciton binding energies compared with aromatic molecules such as PEA, which make (BA)_2_PbI_4_ an interesting candidate for studying exciton-polaritons (E-Ps) within two-dimensional Ruddlesden–Popper perovskite flakes.^53^ We schematically showcase the formation of waveguide modes within exfoliated thin RPP flakes in Figure 1a. Total internal reflection from the boundaries causes the thin crystal to act as a cavity itself, with different cavity modes and their optical dispersion being dependent on the thickness of the flake. Because of the wave function overlap of the excitons and the confined optical modes, they hybridize to polaritonic waveguide modes of which the electric field distribution of the ground mode has been simulated and is depicted in Figure 1a.

An exemplary dispersion relation of these E-Ps is given in Figure 1b, showing the characteristic avoided crossing of the cavity modes with the excitonic resonance, where the dispersion of the latter can be approximated to be constant in low-momentum ranges. Here, the presence of the excitons and the subsequent hybridization cause a bending of the photonic modes away from the excitonic resonance, dividing their dispersion into upper and lower polariton branches (UPB and LPB, respectively). To investigate the energy transfer between different polariton branches, we employ two excitation schemes indicated in Figure 1b. First, by choosing the energy range of the incident light, we selectively excite the first UPB, which transfers its energy to LPBs through radiative decay, where possible radiative emission during this process falls within long wavelengths beyond the detection limit of our detectors. When the LPBs couple directly to light, a detectable photoluminescence (PL) signal is emitted. Second, we excite the highest LPB directly by illuminating the RPP flake with a CW laser with a wavelength of 532 nm, just above the excitonic resonance of 510.8 nm, which then relaxes to LPBs at lower energies. We note that the mere presence of a detectable PL signal in Figure 1c indicates the occurrence of this process. Strikingly, the PL intensity map shows PL signal not only at the focal point of the laser in the center of the flake but also at the edges, indicating E-Ps being excited at the laser spot and propagating as waveguide modes toward the edges where they can couple to light again. Thus, we report the direct observation of the long-range propagation of self-hybridized E-P in thin RPP flakes.

**Figure 2:**
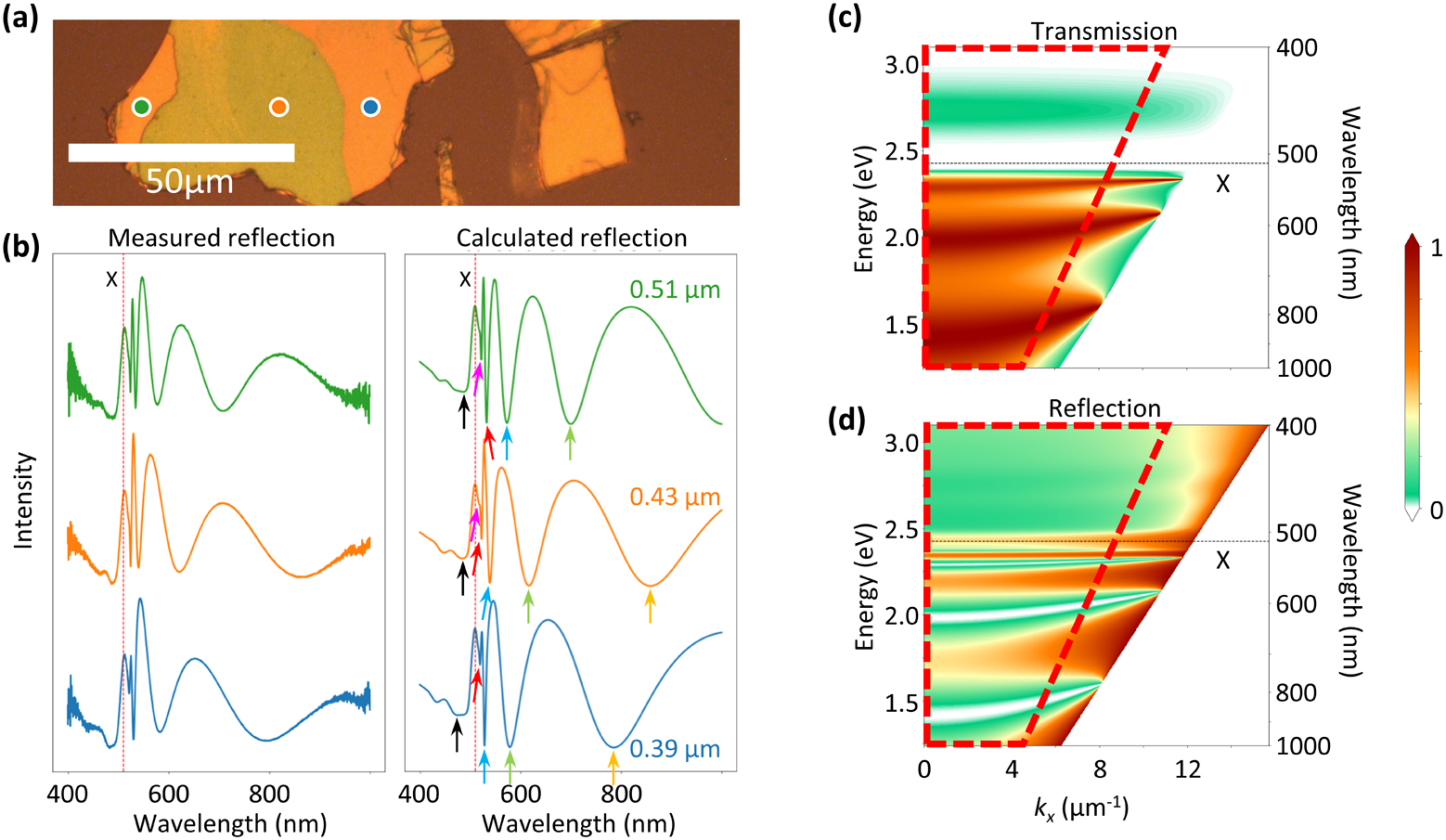
(a) Real-space reflection image of the investigated RPP flake with color-coded positions of the measured reflection spectra. (b) Measured (left) and calculated (right) reflection spectra of the marked positions. The calculations consider the collection angle range of 0 to 44.43° of the microscope objective (NA = 0.7) and are therefore an average over the corresponding in-plane wave numbers *k*_*x*_ = (2π/λ) sin(θ) of the energy-momentum maps, where λ is the wavelength and θ is the incidence angle. The positions are at visibly different heights of the flake and agree perfectly well with the calculated spectra for the selected thicknesses. The arrows mark selected reflectance dips corresponding to Fabry–Pérot resonances. We attribute the dips to the polariton branches UPB_1_ (black), LPB_1_ (red), LPB_2_ (blue), LPB_3_ (green), LPB_4_ (yellow), and a new LPB_1_* (pink), which is emerging with rising thickness. (c) Calculated energy versus momentum transmission and (d) reflection maps for a thin film with a thickness of 430 nm. The red trapezoids mark the area corresponding to the average over the incidence angle range. The Fabry–Pérot resonances bend due to strong coupling with the excitons at the energy marked by the dotted line. The formation of lower and upper polariton branches below and above the exciton energy is observed in both the reflection and the transmission map.

To justify this conclusion in detail, we investigate the formation of polaritons using reflection, transmission, and absorption spectroscopy. The exfoliated flakes show a broad variety of colors dependent on their thickness, which is easily visible in the reflection image in Figure 2a. This directly stems from the Fabry–Pérot resonances occurring in the mesoscale films, causing wavelength-dependent reflection and transmission dips and peaks, respectively. To explore the momentum-dependent reflection, transmission, and absorption spectra of the sample, we utilize a model system composed of a layer of RPP with a known thickness surrounded by a vacuum and solve the wave equation for this system versus the photon energy and the incidence angle. To introduce the nonlinearity of the RPP into this classical calculation, we use measured data of the dielectric function of (BA)_2_PbI_4_ for the RPP layer.^54^ We particularly observe an excellent agreement between the s-polarized incident light in our calculations compared to measured results, as expected due to the mainly in-plane polarization of the light in our measurement schemes.

Because the microscope objective used to measure the spectra in Figure 2b has a numerical aperture of 0.7 and thus a collection angle of 0 to 44.43°, we average our calculations over this angle range and compare the resulting spectra to the measured spectra in Figure 2b. For given values of thickness, we observe remarkable agreement with the measurements, confirming the accuracy of our calculations and allowing conclusions to be drawn about the dispersion relations. In both the reflection and the transmission energy-momentum maps in Figure 2c,d, several cavity modes are evident, showing a clear avoidance of the excitonic resonance and subsequently a bending of their dispersion. Additionally, in all of the reflection spectra and in the calculated reflection energy-momentum maps, a peak coincides with the exciton resonance. Although anomalous dispersion is expected from a standalone absorber, the appearance of a reflection peak at the exciton resonance is characteristic of the absorber interacting with the cavity modes.^42^ Thus, we observe LPBs below the exciton energy as dips and peaks in the transmission and reflection spectra, respectively, with varying degrees of hybridization of the exciton with the cavity modes. The thickness-dependent dispersion of the LPBs is evident from the reflectance dips in Figure 2b, marked by color-coded arrows with a new first LPB (pink) emerging with increasing thickness as another Fabry–Pérot mode approaches the excitonic resonance from above. Moreover, even faint spectral signatures of UPBs above the exciton resonance are observed in the reflection spectra, which are evident in both calculated and measured spectra, whereas in the transmission spectra, they seem to be suppressed. The fact that the LPBs are better observed in the spectroscopy measurements compared to UPBs is due to the strong attenuation of light within the bulk of the material at photon energies higher than the exciton resonance compared to the energies below the exciton resonance. This happens because of the large imaginary part of the permittivity, as already reported elsewhere.^54^

**Figure 3:**
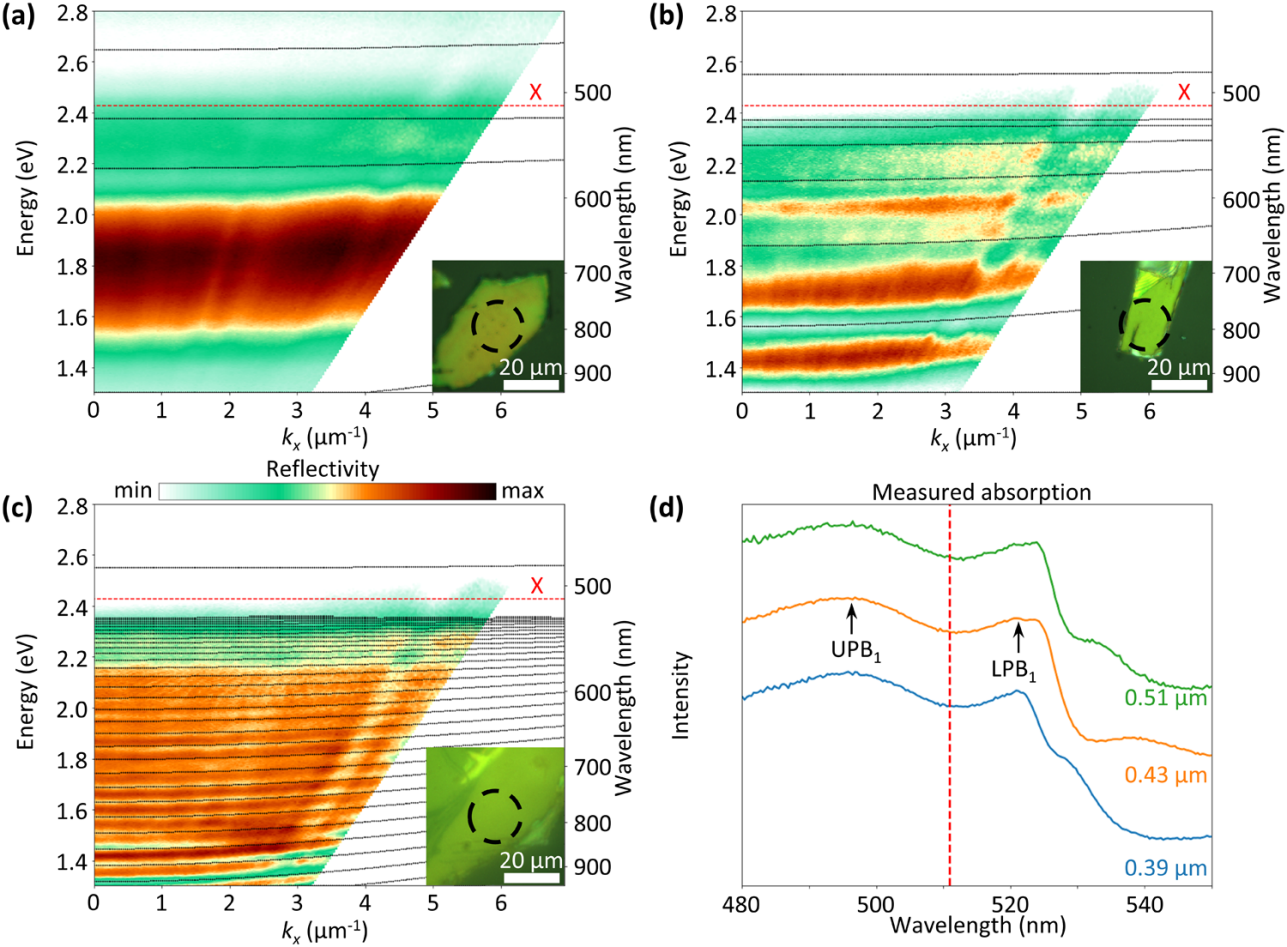
Energy-momentum reflectivity maps of RPP flakes with thicknesses of (a) 246 nm, (b) 770 nm, and (c) 4400 nm. The insets display the real-space reflection images of the corresponding flakes, with the dashed circle indicating the area that contributes to the E-k maps by closing the field stop. The solid lines are dips of the calculated E-k maps similar to those in Figure 2c,d. The visible measured dips are in good agreement with those calculated, both showing bending away from the exciton resonance marked by the red dotted line. Therefore, the dips can be attributed to the diverse multiple branches. (d) Measured absorption corresponding to the color-coded positions and spectra in Figure 2a,b, respectively. The multiple polariton branches appear as peaks above (UPB) and below (LPB) the exciton energy and are linked to those marked in Figure 2b. From the distance between marked UPB_1_ and LPB_1_, the coupling strength for the orange spectrum with a thickness of 430 nm can be calculated. We note that the peaks correspond to the marked dips in Figure 2b.

To experimentally demonstrate the formation of Fabry–Pérot resonances in the RPP films and their strong interactions with excitons in a better way, the angle-resolved reflection spectra of RPP flakes with thicknesses of 246, 770, and 4400 nm are acquired (Figure 3). The changes in the thicknesses are perfectly in correspondence with the number of Fabry–Pérot resonances. Moreover, in all cases, we observe a strong interaction with excitons that leads to a flattening of the Fabry–Pérot branches. The LPBs are excellently captured because of the vanishing imaginary part of the permittivity below the exciton energy, whereas above this energy, the large material dissipation hinders the observation of UPBs.

In these measurements, the beam spot sizes used to excite the flakes are broad to maintain a high spatial coherence level and better *k*-space resolution, where *k* stands for the wavenumber of the light parallel to the plane of the flake. This aspect, however, leads to spot sizes being relatively comparable in size with the sizes of the flakes, as shown in the insets of Figure 3a–c, and thereby, defects and small thickness variations influence the measurement. Our overlaid theoretical calculations of the reflection dips (shown by solid black lines in each panel), where the positions of the dips correspond to the dispersion of the Fabry–Pérot resonances, are qualitatively in good agreement with the measured data. The slight differences in the dispersion of the Fabry–Pérot resonances correspond to the roughness of the flakes, leading to radiation damping of the excitons and slightly lower coupling strength of the excitons to the Fabry–Pérot resonances.

The strong coupling between excitons and Fabry–Pérot resonances of the light captured within the RPP flakes happens at the energy exchange rate between excitons and photons, which is larger than the radiation damping of excitons and photonic modes. Upon such conditions, the splitting of the energy defined as Δω is given by
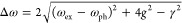
where ω_ex_ and ω_ph_ are the angular frequencies of excitons and photons, respectively; γ is the damping constant; and *g* is the coupling strength.^46^ By considering resonant conditions where ω_ex_ = ω_ph_, the coupling strength is obtained by using the measured absorption spectra that clearly demonstrate the split energy and the formation of the LPB and the UPB. This leads to *ℏg* = 19.9 meV. The damping ratio γ is obtained by fitting a Lorentzian to the data of the dielectric function from the literature.^54^ Δω is sufficiently determined by calculating the measured absorption of the RPP shown in Figure 3d. The polaritonic bands in the E-k maps appear visibly flat near the excitonic energy, supporting the use of the reflectivity data in Figure 2b and the corresponding transmittance data to compute the absorption, which are inherently integrated over a range of incident angles. The measured absorption shows a clear splitting of peaks at the excitonic resonance, varying only slightly with thickness. The orange spectrum exemplifies a Rabi splitting of 120 meV.

**Figure 4:**
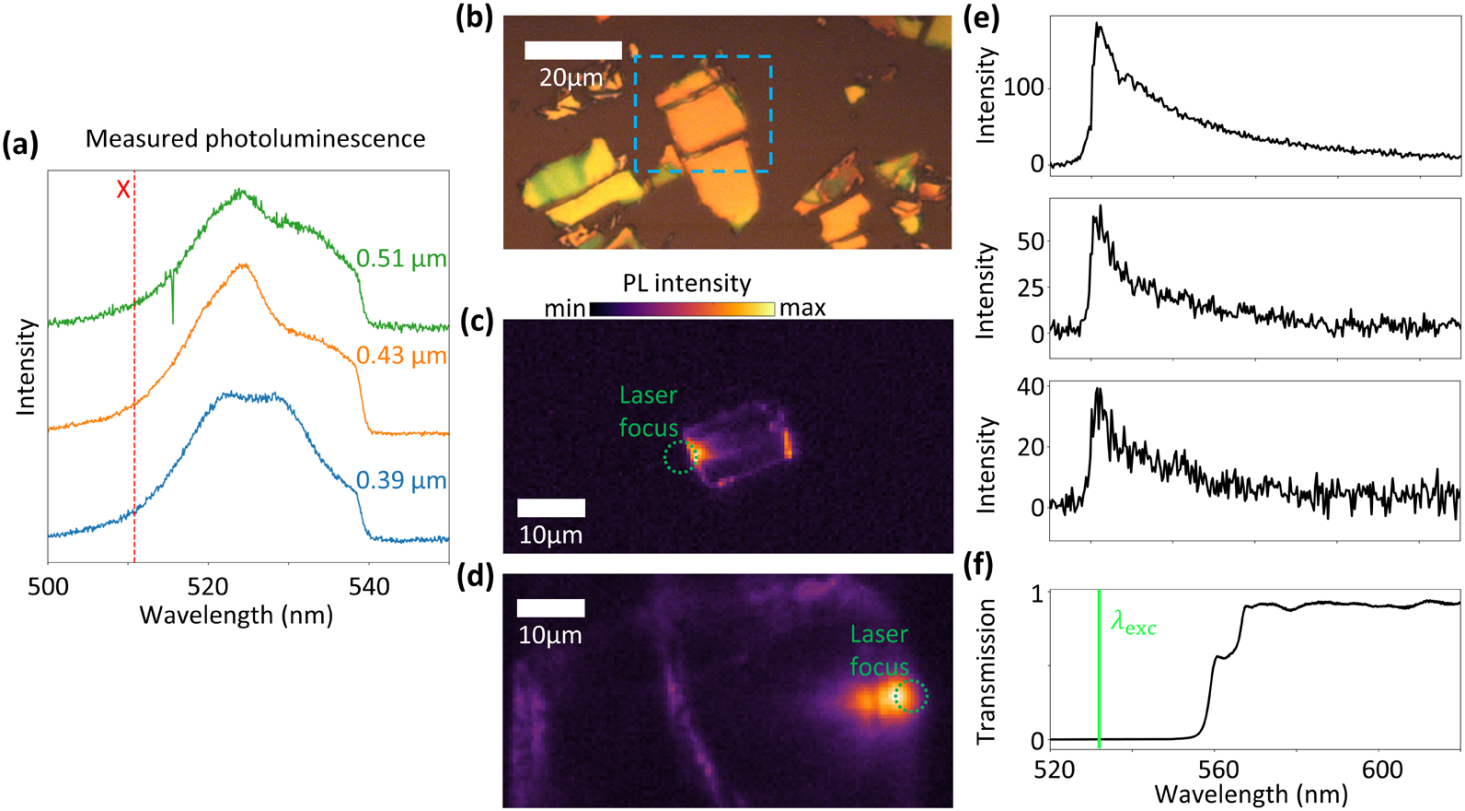
(a) PL spectra of the color-coded positions in Figure 2b acquired by using a 427 ± 15 nm excitation and a 520 ± 17.5 nm emission filter. The spectra show multiple peaks red-shifted with respect to the exciton energy with thickness dependent peak positions that are linked to the marked dips in Figure 2b. (b) Reflection image of the RPP flakes used for the laser-induced PL measurements in panel c. (c) Intensity map of the total PL signal with laser excitation at the edge of the RPP flakes. A clear signal from the edge positioned opposite to the laser focus (green circle) demonstrates the directional propagation of polaritonic waves along the flakes that couple to the light at the edges. (d) PL intensity map of a considerably larger and thicker flake with similar laser excitation. Clear PL signals are visible at a small thickness step on the surface and at the left edge of the flake, indicating waveguided exciton-polaritons having traveled several tens of micrometers. (e) PL spectra corresponding to panel d at the laser focus position (top), the central (center), and the left edge (bottom) with laser excitation at the left edge of the flake. The sharp drop just below 560 nm corresponds to the lowest wavelength that is transmitted by the long-pass filter and dichroic mirror combination used as the emission filter. With laser excitation at 532 nm, the excitons at 511 nm are not excited, but the laser couples directly to an LPB. (e) Transmittance spectrum of the emission filter used for the laser-based PL setup. The spectra in panel d have been corrected by the shape of this transmittance.

To further solidify the conclusion of the polaritonic nature of the observed modes, we acquired the PL spectra of the same positions marked in Figure 2a. The PL spectra in Figure 4a were taken using a band-pass filter with 472 nm center wavelength and 30 nm width in the excitation path and, subsequently, a 520 nm center wavelength and 35 nm width band-pass filter in the emission path; thus, we directly excite in the region of the UPBs. In all three positions, a broad PL signal is observed that is red-shifted with respect to the excitonic resonance, consisting of a superposition of multiple peaks with varying positions depending on the flake thickness. The most intense signal is observed at the highest-energy peak in the blue-colored spectrum, exhibiting the lowest thickness dependence. However, the second peak appears closer to the first and has a similar intensity. We identify the peaks as different LPBs agreeing with the marked dips in the reflectance spectra of Figure 2b, with the first LPB exhibiting the strongest coupling of the cavity mode to the exciton, leading to a pronounced bending of its dispersion. This yields the flattest dispersion and the least sensitivity to thickness for this mode, whereas the energy of lower-energy LPBs experiences a larger dependence on the thickness of the flake. As indicated by the varying intensity, the energy transfer to the LPBs is dependent on the acquisition position. The most efficient transfer occurs from the excited UPB to the first LPB, whereas relaxation to the second LPB becomes more effective at specific thicknesses of the flakes. We note that the excitation scheme allows relaxation from both the UPBs and the first LPB to the second LPB, both contributing to its elevated intensity in the blue spectrum.

The latter process of energy transfer between LPBs is better configured by the PL signal in Figure 4c, where the flake marked in Figure 4b is excited with a 532 nm CW laser focused on its left edge. Because the exciton at 510.8 nm cannot be excited in this way, the observed signal has to stem from one or multiple LPBs. Similar to Figure 1, the excited mode emits light at the position of the laser focus and at the opposing edge. This means that depending on their in-plane momentum component, the exciton-polaritons are guided through the waveguide formed by the RPP flake itself and couple to light at its edge. Additionally, as the edge PL signal is strongest in the direction perpendicular to the edge where the laser spot couples light into the flake, we deduce that the propagation direction is dependent on the shape of the coupling edge. Because the in-plane momentum is expected to determine the efficiency of modes being guided along the flake, the light coupled out at the opposing edge should, on average, be emitted at a higher angle compared to the incidence angle. Therefore, we expect the signal at the opposing edge to be slightly blue-shifted, thus causing the detected tail of the LPB to be of weaker intensity. Remarkably, the total PL signal at the opposite edge is only slightly weaker than that at the laser spot, indicating minimal loss during propagation. Here, the signal at the laser spot includes both radiative Fabry–Pérot modes confined within the light cone and nonradiative Fabry–Pérot modes outside of it. These nonradiative modes primarily couple to light at edges and defects, suggesting that the signal at the opposing edge is dominated by them. To distinguish between radiative and nonradiative modes, we conducted additional measurements on a larger and thicker RPP flake with a small thickness step at its surface, as shown in the PL map in Figure 4d. A pronounced PL signal originating from the laser focus and extending onto the flake surface is attributed to radiative Fabry–Pérot modes, noticeably diminishing well before the edges. Therefore, it is evident that the PL signal at the two consecutive opposing edges stems from nonradiative modes coupling to light at the edges. We note that the PL spectra corresponding to the laser focus and the edges in Figures 1c and 4c,d all show a shape similar to those in Figure 4e, consisting of the tail of an LPB or of the superposition of multiple LPBs cut off by the combination of dielectric mirror and edge-pass emission filter with the latter’s measured transmittance depicted in Figure 4f. Hence, for reasons of clear arrangement, we limit our discussion to Figure 4e, where the spectra show the best signal-to-noise ratio. In this case, the spectrum at the excitation spot (top) is significantly more intense than at the opposing edges, whereas the signals at the first opposing edge (center) are only slightly stronger than at the second edge (bottom) despite being roughly 20 μm apart from each other. We thus conclude that we observe long-range propagating self-hybridized E-Ps with a controllable propagation direction in thin RPP flakes.

**Figure 5:**
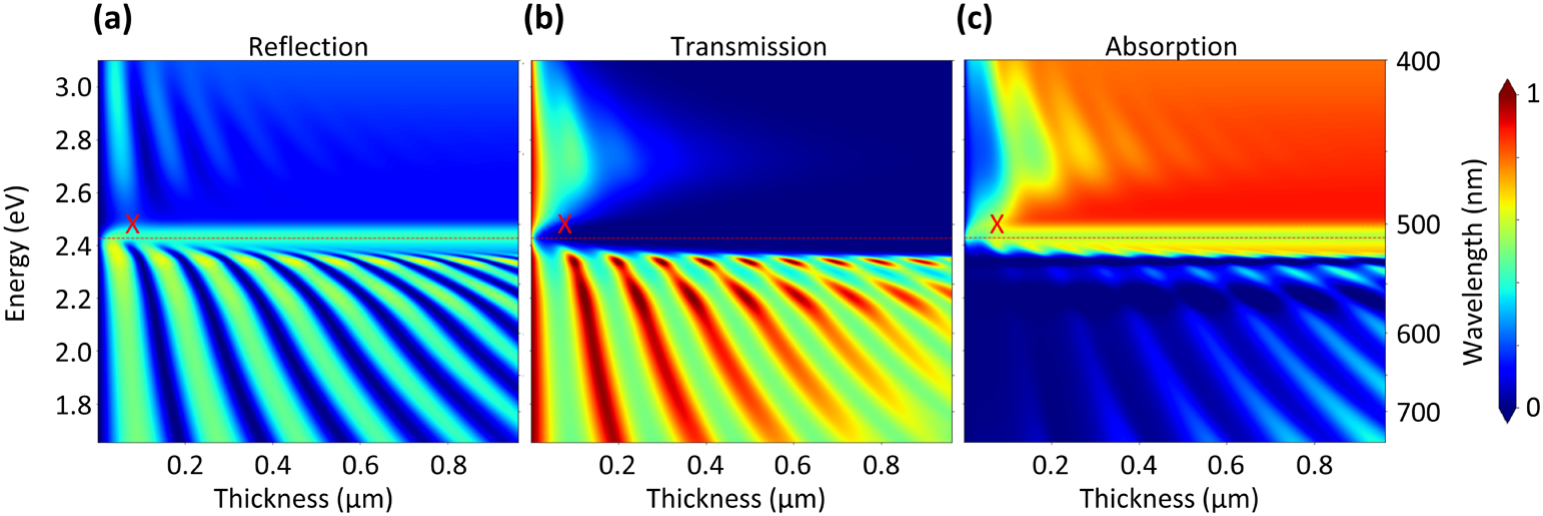
Calculated (a) reflection, (b) transmission, and (c) absorption spectra of thin RPP crystals that are plotted versus the photon energy and crystal thickness. Corresponding to the calculated and measured spectra in Figure 2b, the spectra were averaged over the angle range of 0 to 44.43°. With increasing thickness, multiple Fabry–Pérot resonances appear that show significant bending in the vicinity of the exciton energy marked by the gray line. Remarkably, the absorption spectra show a dip at the exciton energy due to the Rabi splitting in the strong-coupling regime.

Having established the formation of E-Ps in our thin RPP slab waveguides, we briefly address their dependence on the thickness of the flakes. Hence, we plot the spectra calculated in the same fashion as in Figure 2b versus the flake thickness in Figure 5. The resulting thickness-dependent dispersions show polaritonic features that have been discussed previously, such as the reflection peak at the exciton resonance. Moreover, they exhibit polaritonic behavior at every elevated thickness. We see both UPBs and LPBs most clearly in the reflection plot of Figure 5a and the absorption plot in Figure 5c. Their bending is enhanced in the vicinity of the exciton energy, which agrees with the lowered thickness sensitivity of the first LPBs in the PL spectra. Additionally, a permanent dip in the absorption versus thickness plot resides at the exciton energy linked to the polaritonic Rabi splitting.

**Figure 6:**
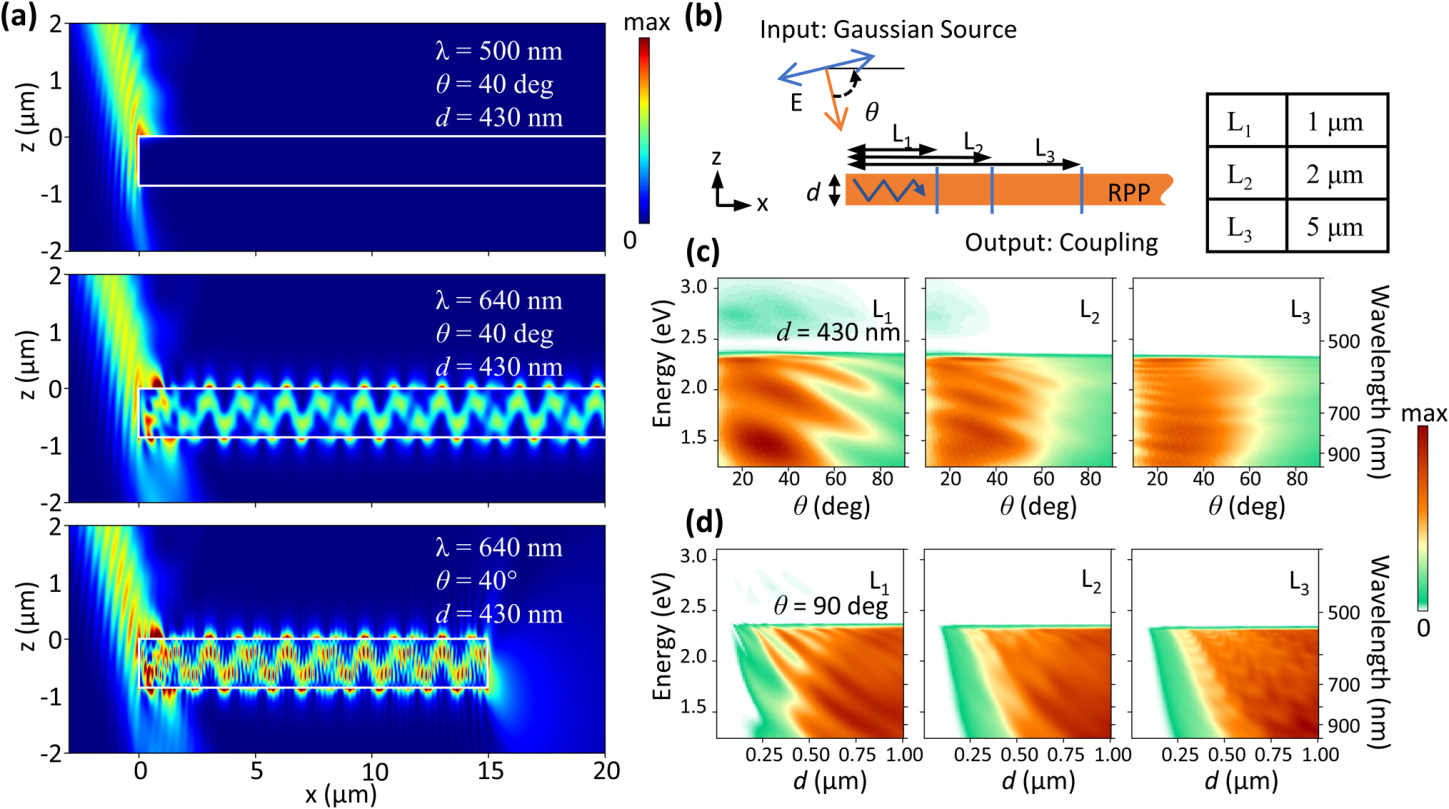
Numerical simulation of the coupling of incident light to the 2D RPP waveguide and subsequent propagation. (a) Cross sections of the electric field distribution of a monochromatic TE-polarized Gaussian beam illuminating a 430 nm thick flake at an angle of θ = 40° with respect to the horizontal axis. The incident beam illuminates the left edge of an infinite flake at respective wavelengths of 500 nm (top) and 640 nm (center) and of a finite flake with a length of 15 μm at a wavelength of 640 nm (bottom). (b) Schematic cross-section view of the illumination geometry with flake thickness *d* and incidence angle θ. Three monitors *L*_1_, *L*_2_, and *L*_3_ are probing the output power at different distances from the left edge, exploring the optical coupling of the beam to waveguide modes and their propagation along the flake infinite in the *x* direction: (c, d) energy-resolved ratio of the output power at the different monitors to the incident input power plotted versus (c) incidence angle θ and (d) thickness *d*.

Complementary to the measured propagation mechanisms of E-Ps, we investigated the coupling efficiency of incident light to E-Ps at the edge of a thin film and the propagation behavior of E-Ps along the RPP flake theoretically. Here, a monochromatic TE-polarized Gaussian beam illuminates the left edge of a 2D RPP film with 430 nm thickness with an incidence angle of 40° with respect to the horizontal axis. We show cross sections of the normalized electric field distribution in Figure 6a. Whereas Figure 5a reveals no coupling at the incident wavelength of 500 nm, a large coupling efficiency into the semi-infinite 2D RPP flake is evident at 640 nm. Moreover, we find a multimode excitation showing nearly lossless propagation along the *x* direction of the infinite flake with the latter owing to the negligible imaginary component of the permittivity at this energy range. With the same parameters and illumination geometry as in the latter case but considering a flake with a finite length of 15 μm, the confined light again efficiently couples out to the radiation continuum at the right edge. This simulation result is in accordance with the observed experimental results in Figure 4, pointing out the long-range propagation of the LPBs and their coupling to light at the opposing edge. The fine interference fringes in the bottom part of Figure 5a are explained by the partial reflection of the guided waves from the right edge.

Furthermore, we investigate the coupling efficiencies and the consequent propagating behavior in a semi-infinite flake by placing three vertical output monitors—*L*_1_, *L*_2_, and *L*_3_—at different distances from the left edge, as indicated by the blue lines in the schematic in Figure 6b. The energy-resolved transmission values at the monitors, achieved by dividing the output optical power by the incident beam power, are plotted versus the incident angle in Figure 6c and versus the flake thickness in Figure 6d. Figure 6c reveals that the optical coupling into the flake occurs for almost all of the incident energies lower than the exciton energy at angles up to about 60°, which can be attributed to the fact that scattering at the edge closes the momentum gap to all possible waveguide modes at nearly all possible in-plane momentum values. Moreover, the optical coupling is observed to be maximized within the angle range of θ = 20–40° for different energies, whereas the coupling strength is diminished for incident angles larger than about 40°. As the coupled wave propagates along the flake, we observe close to no diminishing of the mean power between *L*_1_ and *L*_3_, with the only difference being interference patterns in *L*_1_ caused by the propagating wave overlapping with the incident beam. A similar picture is drawn in Figure 6d. Again, we observe a very low loss between *L*_1_ and *L*_3_. It is evident that as the flake thickness increases, the coupling efficiency increases as well resulting from the emergence of numerous modes that the incident optical beam can be coupled into. Whereas very thin flakes host no cavity modes, the coupling efficiency increases from *d* = 0.25 μm at the energy ranges just below the excitonic resonance because Fabry–Pérot modes appear at lower wavelengths first. Moreover, at shorter distances, where the existence of the UPBs is observed, the energy-splitting caused by the strong interaction between the exciton resonances and the Fabry–Pérot modes is evident. The disappearance of UPBs at longer distances noted by *L*_2_ and *L*_3_ is due to the fact that UPBs cannot propagate long because of the strong attenuation constant of these branches. Comparing this fact with the experimental and theoretical explanations above, the excited waveguiding modes have an exciton-polaritonic nature. This theoretical investigation confirms nearly perfect optical coupling of the incident beam to the E-Ps in the flake at the edges and a nearly lossless propagation at long distances, reinforcing our finding of long-range self-hybridized E-Ps.

## Conclusions

In summary, we have probed the formation and long-range propagation of self-hybridized exciton polaritons in submicrometer thin two-dimensional Ruddlesden–Popper perovskite waveguides by means of transmission, reflection, and photoluminescence spectroscopy. Our experimental results are further supported by theoretical dispersion calculations and by numerical calculations exploring the coupling efficiency of the optical waves into the exciton-polaritons. The slab waveguide formed by the thin Ruddlesden–Popper perovskite crystal yields strong coupling between the cavity modes and excitons. Thus, the ensuing polariton formation modulates the optical response of the material, which is observable in the optical dispersion. This enables the absorption of photons both below and above the band gap into lower and upper polariton branches, respectively, as well as energy transfer between these branches. We directly probe these processes by selecting the exciting photon energy and detecting sub-band-gap emission. Because of the dielectric nature of the Ruddlesden–Popper perovskite below the excitonic energy, the lower polariton branches exhibit highly effective energy transmission via the guided modes across the thin flake with minimal indications of loss. By localized illumination with a subexciton-energy laser, we show the direct excitation of these modes and their relaxation into lower-energy polariton branches, causing a photoluminescence signal at the edges far away from the laser spot. This demonstrates that light can couple to these guided modes either directly or through scattering at the edges, closing a possible in-plane momentum mismatch between incident light and the propagating polariton mode (e.g., at normal incidence). Consequently, this opens up a wide range of excitation schemes, determining the propagation direction not only by the incident light but also by defined structures in the vicinity of the Ruddlesden–Popper perovskite layers. Furthermore, the polariton dispersion is tuned by the flake thickness, contributing to the parameter space used to control the polariton propagation. Thus, our results provide new ways to investigate exciton polaritons in a system with intriguing simplicity and a high degree of versatility, encouraging future research into the characteristics of exciton-polaritons and their associated effective photon–photon interactions.

## Materials and Methods

### Sample Preparation

For the synthesis of the 2D RPP bulk crystal, first *n*-butylammonium iodide (BAI) was synthesized in a separate setting by slowly adding 25 mL of 57% w/w aqueous hydriodic acid (HI) to 5 mL of *n*-butylamine (BA) in an ice bath and stirring for 4 h. Second, 500 mg of PbO powder was dissolved in a mixture of HI solution (57%,3 mL) and H_3_PO_2_ solution (50%,850 μL), while heating at 120 °C and stirring for about 5 min, which led to a bright yellow solution product of PbI_2_. Then, 1.5 mL of the synthesized *n*-CH_3_(CH_2_)_3_NH_3_I (BAI) was added to the PbI_2_ solution, initially producing a black precipitate, which was subsequently dissolved by continued heating at 120 °C. Stirring was stopped after 5 min, and the solution was left to cool to room temperature, during which orange rectangular-shaped crystalline flakes were formed. Then, using vacuum filtering, BA_2_PbI_4_ orange rectangular flakes were separated from the solvent and left to dry for a few days. After that, the flakes were deposited on a substrate by mechanical exfoliation where a flake was picked up by a piece of Scotch brand tape, and after folding and unfolding several times, several flakes with reduced thicknesses to hundreds of nanometers were achieved that then were stamped on the substrate.

### Optical Spectroscopy

Reflectance and transmittance were recorded from the RPP flakes exfoliated on glass using a Nikon Eclipse Ti2-A inverted microscope and its built-in halogen lamps in an assembled system provided by New Technologies and Consulting Company, and the angle-resolved reflectance was measured on a similar system with a Nikon Eclipse Ti inverted microscope. All were normalized by dividing through the reflectance of a silver mirror and the transmittance of glass, respectively. The photoluminescence measurements were carried out with a halogen lamp, filtered by a 472 ± 15 nm excitation filter, and with a 532 nm CW laser. The low power of the halogen lamp within the excitation band proved to be sufficient for preventing photobleaching of the perovskites, and for the same reason, the laser power was damped by neutral density filters. The PL measurements were performed in such a way as to maintain the stability of RPP flakes upon illumination with light beams. Perovskites including RPP are well-known to suffer from photoinduced bleaching. Therefore, the measurements have to be performed within short acquisition times to avoid degradation, thereby leading to a lower signal-to-noise ratio (Figure 4d). To separate the excitation illumination, which was incident from the optical reflection path, from the emission signal, dichroic mirrors were employed in combination with bandpass and edgepass filters. The spatially resolved optical spectra were measured in a Princeton Instruments SpectraPro HRS 500-S grating spectrometer using a reflective grating with 150 grooves/mm for the reflection/absorption, whereas for photoluminescence measurements, a grating with 600 grooves/mm was utilized. The angle-resolved reflectivity spectra were recorded by projecting the back focal plane, also called the Fourier plane, onto the slit of the spectrometer by an additional lens. For this, a Princeton Instruments IsoPlane 160 spectrometer equipped with a 300 grooves/mm grating was used. All measurements were performed at room temperature.

### Analytical and Numerical Calculations

For the analytical dispersion calculation, a layered model was defined with infinite extension in the *x* and *y* direction, which consists of a vacuum, a material with the relative permittivity ε_r_(ω) of RPP, and then a vacuum again, all arranged along the *z* direction in the sequential order. With light incident from the positive *z* direction, the propagation is calculated in the *x* direction, and the permittivity is assumed to be isotropic, which yields
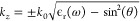
Here, *k*_*z*_ is the in-plane wavenumber of the propagating wave, *k*_0_ denotes the wavenumber of the incoming radiation, and θ is its angle of incidence. Defining *z*_1_ = *d* and *z*_2_ = −*d* as the positions of the boundaries, the wave equation of for s-polarized incident light yielding the time-harmonic solution of the wave equation becomes
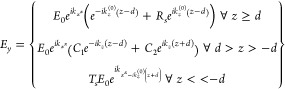
with reflection amplitude *R*_S_, transmission amplitude *T*_S_, and the constant amplitudes *C*_1_ and *C*_2_. A similar expression for *H*_*x*_ is found by , and applying the boundary conditions that *E*_*y*_ and  have to be continuous at *z = d* and *z = −d* results in the following equation system:
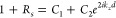

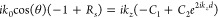

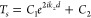

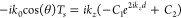


The solutions for *R*_S_ and *T*_S_ represent reflectance |*R*_S_|^2^ and transmittance |*T*_S_|^2^. It should be noted that a plane wave focused by the microscope objective can interfere with itself. Hence, taking the averages of *R*_S_ and *T*_S_ is done before taking their absolute values when calculating the averaged reflectance and transmittance over the corresponding range of incident angles.

In the case of the simulations of the coupling and propagation efficiency, the finite difference time domain (FDTD) was utilized as the numerical method. Perfectly matched layers (PMLs) were used as the boundary conditions at the boundaries of the simulated box for which Maxwell’s equations have been solved. Here, a TE-polarized beam was incident at the edge of an RPP layer with semi-infinite or finite extension in the *x* direction and infinite extension in the *y* direction, and the resulting electric field distribution was calculated.

## Notes

The preprint version of our work in this manuscript is available online.^55^
